# Online Dignity Therapy and Photo Collages: The Narratives of Israeli and Italian Older Adults in the COVID-19 Era

**DOI:** 10.1093/geroni/igab046.2169

**Published:** 2021-12-17

**Authors:** Shoshi Keisari, Talia Elkarif, Giada Mola, Ines Testoni, Silvia Piol

**Affiliations:** 1 University of Haifa, Department of Gerontology, Faculty of Social Welfare and Health sciences, Mount Carmel, HaZafon, Israel; 2 University of Haifa, School of Creative Arts Therapies, Faculty of Social Welfare and Health Sciences, Mount Carmel, HaZafon, Israel; 3 Università degli studi di padova, DPSS Department, padova, Veneto, Italy; 4 Università degli studi di padova, FISPPA Department, Section of Applied Psychology, Padova, Veneto, Italy; 5 Università degli Studi di Padova, Padova, Veneto, Italy

## Abstract

The social isolation imposed by the Covid-19 pandemic has significantly affected older adults, and has impacted both their physical and mental health. The pandemic has led to an increase in ageism associated with poorer mental health and a lower sense of dignity, self-esteem and contribution to society. This cross-cultural study involved 24 participants from Italy and Israel aged 79 to 92. The aim was to develop a brief art-based online intervention to enhance the participants’ sense of dignity and sense of meaning in life during this time of crisis. The process focused on the creation of digital photo-collages that captured the participants’ values through three perspectives: their past experiences, legacy, and future perspectives. It employed an arts-based research methodology to explore the participants’ experiences by analysing their relationship with the artistic expression, the photo collage, and its creative process.

